# Complete mitochondrial genome of *Ctenophthalmus quadratus* and *Stenischia humilis* in China provides insights into fleas phylogeny

**DOI:** 10.3389/fvets.2023.1255017

**Published:** 2023-09-13

**Authors:** Bin Chen, Ya-fang Liu, Xin-yan Lu, Dan-dan Jiang, Xuan Wang, Quan-fu Zhang, Guo-ping Yang, Xing Yang

**Affiliations:** ^1^Integrated Laboratory of Pathogenic Biology, College of Preclinical Medicine, Dali University, Dali, China; ^2^School of Public Health, Dali University, Dali, China; ^3^Nanchang University Queen Mary School, Nanchang University, Nanchang, China; ^4^Department of Gastroenterology, Clinical Medical College and the First Affiliated Hospital of Chengdu Medical College, Chengdu, China

**Keywords:** *Ctenophthalmus quadratus*, *Stenischia humilis*, mitochondrial genome, phylogenetic analyzes, fleas

## Abstract

Fleas (Order Siphonaptera) are common blood-feeding ectoparasites, which have important economic significance. Limited mitochondrial genome information has impeded the study of flea biology, population genetics and phylogenetics. The *Ctenophthalmus quadratus* and *Stenischia humilis* complete mt genomes are described in this study. The samples were collected from Jianchuan, Yunnan plague foci, China. The mt genomes of *C. quadratus* and *S. humilis* were 15,938 bp and 15,617 bp, respectively. The gene arrangement of mt genome was consistent with that of other fleas, which include 22 tRNA genes, 13 protein-coding genes, and two rRNA genes, with a total of 37 genes. The relationship between *C. quadratus* and *S. humilis* in fleas was inferred by phylogenetic analysis of mt genome sequence datasets. Phylogenetic analyzes showed that the *C. quadratus* and *S. humilis* belonged to different species in the same family, and were closely related to *Hystrichopsylla weida qinlingensis* in the same family; and revealed that the family Hystrichopsyllidae is paraphyletic, supporting the monophyly of the order Siphonaptera. This study decodes the complete mt genomes of the *C. quadratus* and *S. humilis* for the first time. The results demonstrate that the *C. quadratus* and *S. humilis* are distinct species, and fleas are monophyletic. Analysis of mt genome provides novel molecular data for further studying the phylogeny and evolution of fleas.

## Introduction

Fleas (Order Siphonaptera) include about 2,574 species in 238 genera and 16 families, are some of the most common blood-feeding ectoparasites of birds and mammals (1). They are the most economically significant ectoparasites, resulting in spending of more than $15 billion per year in the world to control and prevent of flea infestations in companion animals (2). Flea is of major epidemiological importance because they can transmit various pathogens. They have worldwide distribution and a wide range of host preference, and are vectors for many pathogens, such as *Yersinia pestis* (plague), *Rickettsia* spp. (*Rickettsia typhi*, *Rickettsia prowazekii*, and *Rickettsia rickettsi*), and *Bartonella henselae* (cat-scratch disease) (3–5). Plague is the most serious of these diseases, with multiple outbreaks around the world killing hundreds of millions of people (4). Up to now, China has identified 15 natural plague foci covering an area of more than 1.4 million square kilometers (6). The world’s third plague pandemic originated in Yunnan Province, China (7, 8). *Ctenophthalmus quadratus* and *Stenischia humilis* are one of the most common fleas in the focus in Yunnan Province, and *Yersinia pestis* has been isolated from these two fleas many times (9). *Ctenophthalmus quadratus* was considered to be the main vector species of plague bacilli, but later experiments showed that it has no vector role (10).

In fundamental research on these important ectoparasites and the diagnosis of the diseases they transmit, accurate classification and identification of fleas are required (11–13). Due to the absence of molecular data, most fleas are identified solely by their essential shape and morphological identification such as the distribution of their setae, spines, and ctenidia (14). However, the morphological identification of related species and variant species has some limitations and is easy to be misidentified (11). Until now, the phylogenetic relationship of fleas has been unclear, and the phylogeny at high taxonomic levels has been controversial (15). With the development of genetic technology, molecular biological methods have been widely used in taxonomy, population genetics, and systematics, to some extent, to supplement the limitations of traditional morphology (13, 16). The mitochondrial (mt) genomes have been extensively used in molecular phylogenetic studies, genetic diversity, subspecies and cryptic species identification of different ectoparasites at various taxonomic levels because of their rapid evolutionary rate, simple structure, maternal inheritance, high mutation rate, and the lack of genetic recombination (17–21). Nevertheless, there is little information about the whole mt genome of the flea, no more than 10 species, which greatly limits the studies on flea genetics and phylogenetics. Hence, more flea mt genomes need to be investigated to obtain more genetic data.

At present, no molecular data are available for the mt genomes of *Ctenophthalmus quadratus* and *Stenischia humilis*. In this study, the whole mt genome of these two fleas was annotated and analyzed. The intentions of this study were to: (i) sequence and annotate the complete mitochondrial genomes of *C. quadratus* and *S. humilis*; (ii) analyze and compare the structural features of mt genomes of *C. quadratus* and *S. humilis*; and (iii) establish phylogenetic relationships with other fleas to reassess the taxonomic status of *C. quadratus* and *S. humilis* in Yunnan, China.

## Materials and methods

### Sample collection and DNA extraction

Adults of *Ctenophthalmus quadratus* and *Stenischia humilis* were collected from the *Eothenomys miletus* in Jianchuan plague foci (26°12′N, 99°33′E), Yunnan Province, China. The key points of morphological identification of *Ctenophthalmus quadratus* and *Stenischia humilis* were recorded in detail in “the Siphonaptera of Yunnan” (22). The fleas were photographed with the Ultra-Depth Three-Dimensional Microscope (VHX-5000), and the basic morphological features were identified by professionals (22). Then, those identified as *C. quadratus* and *S. humilis* were stored, respectively, in 75% ethanol and stored at −20°C until use. According to the manufacturer’s instructions, the total genomic DNA was extracted from a single intact female flea individual using the QIAamp DNA Mini Kit (Qiagen, Hilden, Germany). The voucher specimen and genome DNA were deposited at the Parasitological Museum, Dali University, Yunnan, China.

### DNA amplification and sequencing

The mitochondrial genomes of *C. quadratus* and *S. humilis* were amplified using designed long-PCR primers based on the *12S rRNA* and *cox1* genes of *Ceratophyllus wui* (MG886872) and *Hystrichopsylla weida qinlingensis* (MH259703) using the Primer3 (23), respectively (Table 1). All PCR reactions (volume: 50 μL) were performed in 23 μL ddH_2_O, 4 μL of dNTP mixture, 4 μL of DNA sample, 4 μL of forward primer, 4 μL of reverse primer, 10 μL of 5X PrimeSTAR GXL Buffer, and 1 μL of PrimeSTAR GXL DNA Polymerase (Takara, Japan). The PCR conditions were as follows: 94°C for 2 min, followed by 35 cycles of 98°C for 10 s, 65–68°C for 30 s, 68°C for 10 min, and with a final extension to 10 min at 68°C. The PCR products were detected in a 1.2% agarose gel electrophoresis upon ethidium-bromide staining. The purified PCR amplicons were sequenced on the Illumina NovaSeq 6,000 platform (Harbin Botai Biotechnology Co., Ltd., Heilongjiang, China).

**Table 1:** The primer sequences used to PCR amplification.

Primer	Sequence
CQ1F	5′-ACTGCACCTTGATTTGACATCT−3′
CQ1R	5′-CCTGTTCTTTAATCGATATTCCACG-3′
CQ2F	5′-GCTTACTTCACCTCAGCTACAATAA-3′
CQ2R	5′-GCCAGTAAATAAAGGGAATCAGTGAAC-3′
SH1F	5′-AACTGCACCTTGATTTGACA-3′
SH1R	5′-CCTGTTCTATAATCGATATTCCACG-3′
SH2F	5′-CCCTGAAGTTTATATCCTAATTCTCCC-3′
SH2R	5′-GGGTGTCAACATCTATTCCAACAGTAA-3′

### Genome annotation

The raw data obtained by sequencing was filtered by AdapterRemoval software (v2.0) to remove the presence of low-quality data. The software FastQC was used to conduct quality control on the clean data filtered in the previous step. Genome assembly was carried out from clean data after sample quality control, and IDBA software was used for assembly (24). The predicted genes were compared with each functional database by BLAST (blastp, evalue ≤1e-5), and the comparison results with the highest score were selected (25). The MITOS webserver was used to annotate the mitochondrial genome (26). The tRNAscan-SE webserver was used to verify transfer RNA (tRNA) genes with secondary structure. The GC skew and AT skew were calculated by the strand asymmetry formulas (27). The amino acid sequences of PCGs, nucleotide composition, and base composition were analyzed using MEGA X (28).

### Phylogenetic analysis

For the phylogenetic relationship analysis, 15 additional mitochondrial genome sequences were downloaded from GenBank, and *Philaenus spumarius* (GenBank accession number: AY630340) was selected as an outgroup (Table 2). The sequences of amino acids of 13 protein-coding genes (PCGs) were aligned using MAFFT software. All positions containing blank and missing data were eliminated. The General Time Reversible (GTR + G + I) model was selected as the most suitable model of evolution by the MrModeltest 2.3 based on the Akaike information criterion (AIC) (29). The Bayesian inference (BI) phylogenetic tree was reconstructed with 1,000,000 generations and sampled every 100 generations in MrBayes 3.2.5 (30). And the maximum likelihood (ML) phylogenetic tree was constructed on IQ-TREE based on 10,000 ultrafast bootstrap approximations (31). The resulting phylogenetic tree was edited using FigTree v.1.4.2.

**Table 2:** The flea species analyzed in the current study with their GenBank numbers.

Species	Family	Length (bp)	GenBank number
*Jellisonia amadoi*	Ceratophyllidae	17,031	KF322091
*Ceratophyllus wui*	Ceratophyllidae	18,081	MG886872
*Dorcadia ioffi*	Vermipsyllidae	16,785	MF124314
*Hystrichopsylla weida qinlingensis*	Hystrichopsyllidae	17,173	MH259703
*Ctenocephalides felis felis*	Pulicidae	20,911	MW420044
*Ctenocephalides canis*	Pulicidae	15,609	ON109770
*Xenopsylla cheopis*	Pulicidae	18,902	MW310242
*Pulex irritans*	Pulicidae	20,337	ON100828
*Panorpa debilis*	Panorpidae	17,018	MK870081
*Neopanorpa pulchra*	Panorpidae	15,531	FJ169955
*Cerapanorpa obtusa*	Panorpidae	16,318	KX091860
*Neopanorpa chelata*	Panorpidae	16,342	KX091857
*Bittacus pilicornis*	Bittacidae	15,842	HQ696578
*Bittacus strigosus*	Bittacidae	15,825	MK870080
*Bittacus planus*	Bittacidae	15,031	KX091849

## Results

### General characteristics of the mitochondrial genomes

**Figure 1:**
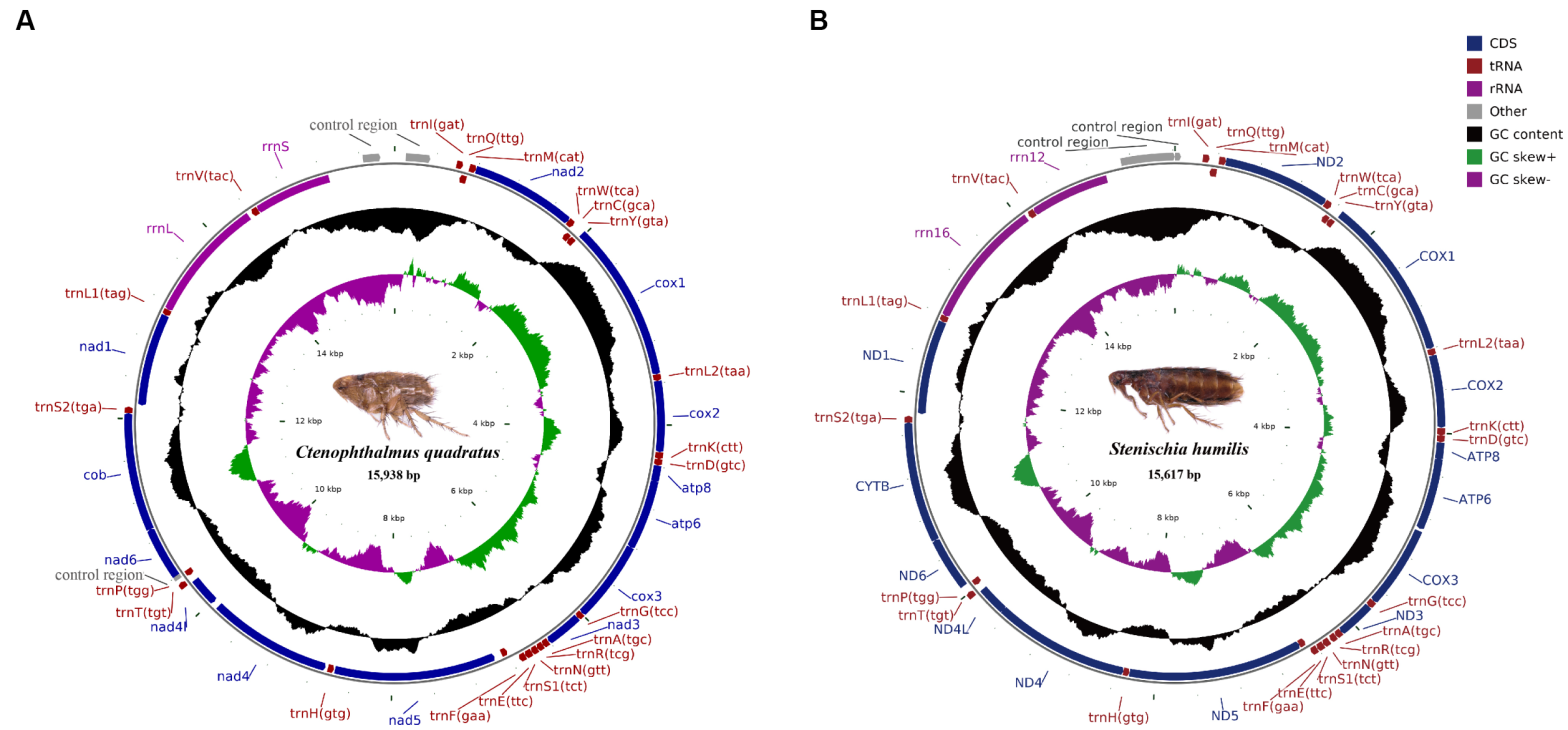
Arrangement of the mitochondrial genome of **(A)** *Ctenophthalmus quadratus* and **(B)** *Stenischia humilis*. All genes are indicated using standard nomenclature.

The complete mitochondrial genomes of *Ctenophthalmus quadratus* and *Stenischia humilis* were submitted to GenBank with accession numbers OQ023577 and OQ366410. The sequences of *C. quadratus* and *S. humilis* was 15,938 bp and 15,617 bp in size, respectively, and both contained 37 genes, including 22 tRNA genes, 13 PCGs, and two rRNA genes (Figure 1). 23 genes (nad2, cox1, cox2, atp8, atp6, cox3, nad3, nad6, cob, trnI, trnM, trnW, trnL2, trnK, trnD, trnG, trnA, trnR, trnN, trnS1, trnE, trnT, trnS2) were on the heavy strand (H-strand), while the rest 14 genes (nad5, nad4, nad4L, nad1, trnQ, trnC, trnY, trnF, trnH, trnP, trnL1, trnV, rrnL, rrnS) were on the light strand (L-strand). The mitochondrial genome of *C. quadratus* had 13 overlapping locations, consisting of a total of 36 bp, with each overlap one to eight bp, and had seven intergenic regions comprising 151 bp in total and ranging from one to 73 bp (Table 3). Likewise, the mt genome of *S. humilis* had 11 overlapping locations with a total length of 33 bp, and had 16 intergenic regions consisting of 183 bp in total (Table 3). The nucleotide composition of *C. quadratus* was: A = 39.19%, T = 40.26%, G = 7.96%, C = 12.59%, and A + T = 79.45% (Table 4). Similarly, The nucleotide composition of *S. humilis* was: A = 38.57%, T = 39.43%, G = 8.38%, C = 13.62%, and A + T = 78.00% (Table 4).

**Table 3:** Organization of the mitochondrial genomes of *Ctenophthalmus quadratus* and *Stenischia humilis*.

Gene	Strand	Positions	Size (bp)	Initiation codon	Termination codon	Anticodon	Intergenic nucleotides
trnI	H	596–657/262–324	62/63			GAT	
trnQ	L	723–655/409–341	69/69			TTG	-3/16
trnM	H	723–788/415–480	66/66			CAT	−1/5
nad2	H	789–1,799/481–1,485	1,011/1,005	ATT/ATT	TAA/TAA		0/0
trnW	H	1,799-1,862/1,487-1,552	64/66			TCA	-1/1
trnC	L	1,915-1,855/1,605-1,545	61/61			GCA	-8/−8
trnY	L	1,980-1,916/1,668-1,606	65/63			GTA	0/0
cox1	H	1,978-3,516/1,666-3,201	1,539/1,536	ATC/ATC	TAA/TAA		-3/59
trnL2	H	3,517-3,579/3,205-3,267	63/63			TAA	0/3
cox2	H	3,580-4,260/3,268-3,948	681/681	ATT/ATG	TAA/TAA		0/0
trnK	H	4,261-4,327/3,951-4,020	67/70			CTT	0/2
trnD	H	4,327-4,388/4,020-4,085	62/66			GTC	-1/−1
atp8	H	4,389-4,547/4,086-4,247	159/162	ATC/ATT	TAA/TAA		0/0
atp6	H	4,541-5,212/4,241-4,915	672/675	ATG/ATG	TAA/TAA		−7/−7
cox3	H	5,212-5,994/4,938-5,720	783/783	ATG/ATG	TAA/TAA		−1/22
trnG	H	5,995-6,054/5,721-5,787	60/67			TCC	0/0
nad3	H	6,055-6,408/5,788-6,138	354/351	ATA/ATC	TAA/TAG		0/0
trnA	H	6,413-6,475/6,137-6,199	63/63			TGC	4/−2
trnR	H	6,475-6,536/6,201-6,261	62/61			TCG	−1/1
trnN	H	6,537-6,600/6,276-6,340	64/65			GTT	0/14
trnS1	H	6,601-6,669/6,341-6,408	69/68			TCT	0/0
trnE	H	6,670-6,733/6,411-6,475	64/65			TTC	0/2
trnF	L	6,868-6,807/6,537-6,474	62/64			GAA	73/−2
nad5	L	8,582-6,869/8,255-6,537	1,714/1,719	ATA/ATG	T(AA)/TAA		0/−1
trnH	L	8,645-8,583/8,318-8,257	63/62			GTG	0/1
nad4	L	9,981-8,646/9,655-8,318	1,336/1,338	ATG/ATG	T(AA)/TAA		0/−1
nad4l	L	10,268-9,975/9,942-9,649	294/294	ATG/ATG	TAA/TAG		−7/−7
trnT	H	10,271 − 10,334/9,945 − 10,009	64/65			TGT	2/2
trnP	L	10,397 − 10,335/10,073-10,010	63/64			TGG	0/0
nad6	H	10,409-10,918/10,082-10,588	510/507	ATT/ATT	TAA/TAA		11/8
cob	H	10,918-12,054/10,588-11,721	1,137/1,134	ATG/ATG	TAG/TAG		−1/−1
trnS2	H	12,054-12,115/11,720-11,783	62/64			TGA	-1/−2
nad1	L	13,069-12,134/12,734-11,802	936/933	ATG/ATG	TAA/TAA		18/18
trnL1	L	13,132-13,071/12,797-12,736	62/62			TAG	1/1
rrnL	L	14,382-13,133/14,063-12,798	1,250/1,266				0/0
trnV	L	14,492-14,425/14,158-14,092	68/67			TAC	42/28
rrnS	L	15,274-14,492/14,942-14,158	783/785				-1/−1
D-loop		106–314;15,635-15,802/ 1–56;15,110-15,617	404/564				

**Table 4:** Composition of mitochondrial genomes in the *Ctenophthalmus quadratus* and *Stenischia humilis*.

Region	A%	T%	G%	C%	A + T%	G + C%	AT skew	GC skew
Whole genome	39.19/38.57	40.26/39.43	7.96/8.38	12.59/13.62	79.45/78.00	20.55/22.00	−0.014/−0.011	−0.226/−0.238
nad2	35.01/34.43	47.58/47.06	7.12/7.36	10.29/11.14	82.59/81.49	17.41/18.51	−0.152/−0.155	−0.182/−0.204
cox1	30.34/29.82	40.81/39.45	14.04/14.19	14.81/16.54	71.15/69.27	28.85/30.73	−0.147/−0.139	−0.027/−0.076
cox2	35.10/34.65	40.82/38.18	9.99/10.57	14.10/16.59	75.92/72.83	24.08/27.17	−0.075/−0.048	−0.071/−0.222
atp8	39.62/40.12	47.17/44.44	3.77/4.32	9.43/11.11	86.79/84.57	13.21/15.43	−0.087/−0.051	−0.429/−0.440
atp6	34.52/32.59	42.56/41.78	9.23/10.52	13.69/15.11	77.08/74.37	22.92/25.63	−0.104/−0.124	−0.195/−0.179
cox3	29.89/29.12	43.17/41.12	12.39/13.92	14.56/15.84	73.05/70.24	26.95/29.76	−0.182/−0.171	−0.081/−0.064
nad3	34.18/30.77	45.76/46.15	8.47/8.55	11.58/14.53	79.94/76.92	20.06/23.08	−0.145/−0.200	−0.155/−0.259
nad5	35.59/34.44	44.63/44.50	13.13/13.85	6.65/7.21	80.22/78.94	19.78/21.06	−0.113/−0.127	0.328/0.315
nad4	33.23/33.03	46.33/44.69	13.17/14.80	7.26/7.47	79.57/77.73	20.43/22.27	−0.165/−0.150	0.289/0.329
nad4l	33.67/36.05	50.00/47.62	13.61/13.27	2.72/3.06	83.67/83.67	16.33/16.33	−0.195/−0.138	0.667/0.625
nad6	38.24/36.09	44.71/49.51	5.88/4.93	11.18/9.47	82.94/85.60	17.06/14.40	−0.078/−0.157	−0.310/−0.315
cob	30.96/31.22	43.45/40.65	10.47/11.46	15.13/16.67	74.41/71.87	25.59/28.13	−0.168/−0.131	−0.182/−0.185
nad1	33.23/29.80	45.73/46.20	14.21/16.61	6.84/7.40	78.95/75.99	21.05/24.01	−0.158/−0.216	0.350/0.384
rrnL	39.68/40.21	41.84/41.31	12.48/12.72	6.00/5.77	81.52/81.52	18.48/18.48	−0.026/−0.014	0.351/0.376
rrnS	39.59/40.13	41.12/40.89	12.90/12.74	6.39/6.24	80.72/81.02	19.28/18.98	−0.019/−0.009	0.338/0.342
tRNAs	40.71/40.59	39.57/39.12	11.10/11.94	8.61/8.36	80.28/79.71	19.72/20.29	0.014/0.019	0.126/0.176

### Annotation

**Figure 2:**
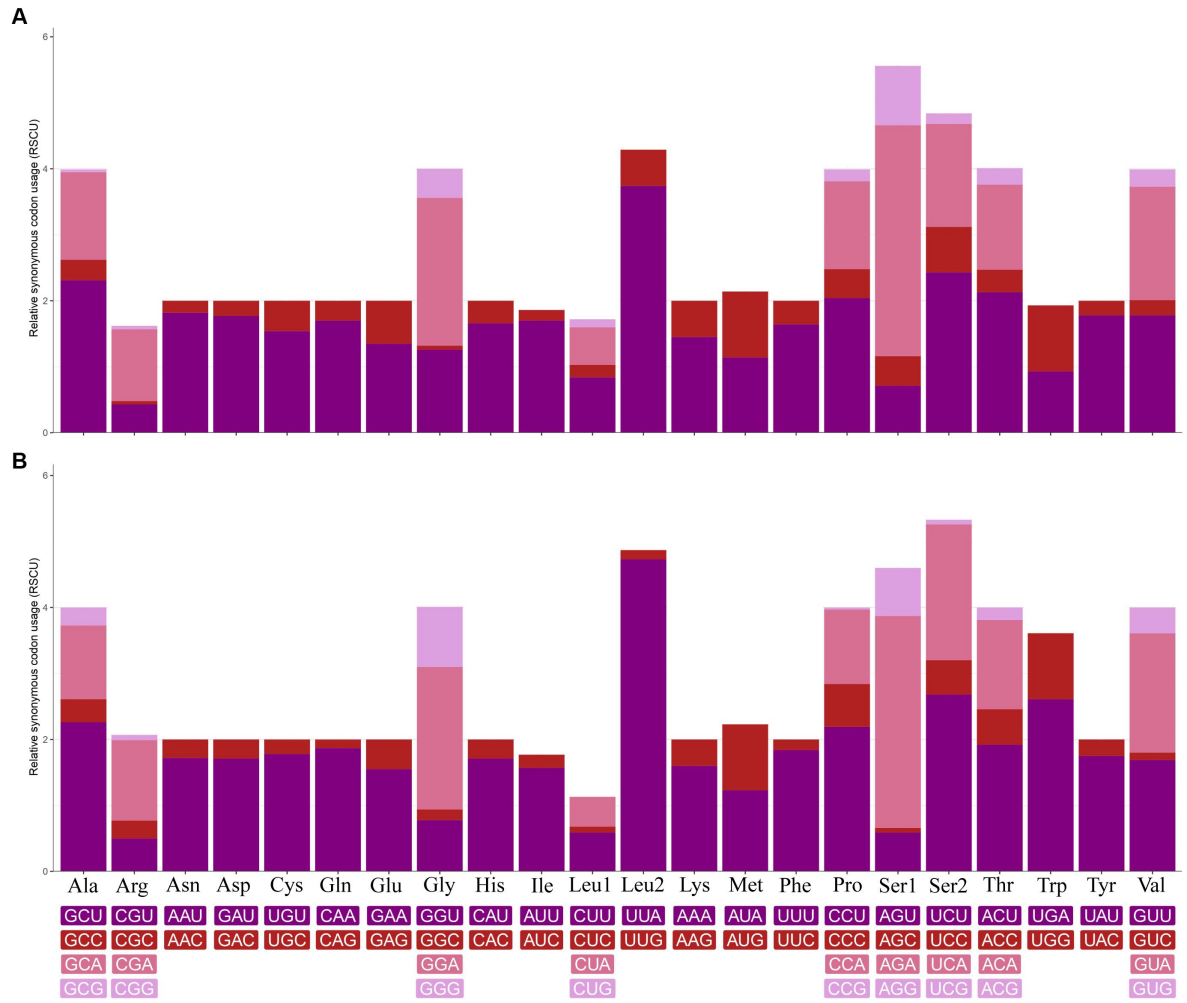
Relative synonymous codon usage (RSCU) of **(A)** *Ctenophthalmus quadratus* and **(B)** *Stenischia humilis*. All codons coding for each amino acid are represented in the boxes below the bar chart.

In the mt genome of *C. quadratus*, three genes (nad2, cox2, nad6) use ATT, two genes (cox1, atp8) use ATC, two genes (nad3, nad5) use ATA, and six genes (atp6, cox3, nad4, nad4L, cob, nad1) use ATG as initiation codon, respectively, and TAA as termination codon in addition to nad5, nad4 and cob genes (Table 3). In the *S. humilis* mt genome, three genes (nad2, atp8, nad6) use ATT, two genes (cox1, nad3) uses ATC and eight genes (cox2, atp6, cox3, nad5, nad4, nad4L, cob, nad1) use ATG as initiation codon, respectively, and TAA as termination codon except for nad3, nad4L and cob genes (Table 3). The relative synonymous codon usage (RSCU) values and codon use patterns were computed for 13 protein-coding genes of the *C. quadratus* and *S. humilis* mt genomes. In total, 3,546 and 3,693 amino acids were encoded by the mt genomes of *C. quadratus* and *S. humilis*, respectively. Leucine, isoleucine, and phenylalanine were the most frequently utilized amino acids, followed by serine and methionine; arginine and cysteine were the least utilized amino acids (Figure 2).

**Figure 3:**
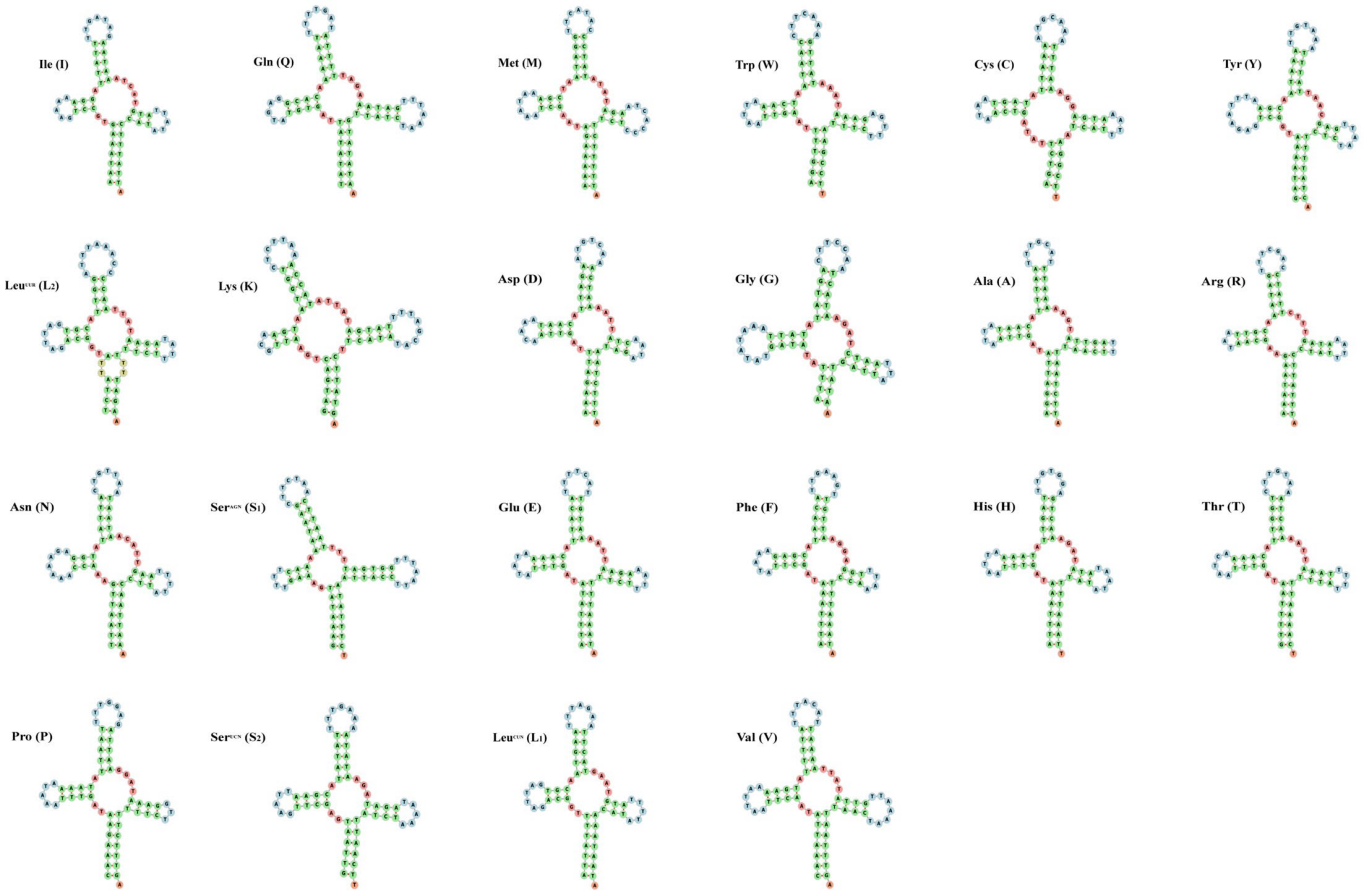
Putative secondary structure of the 22 mt tRNA of *Ctenophthalmus quadratus*.

**Figure 4:**
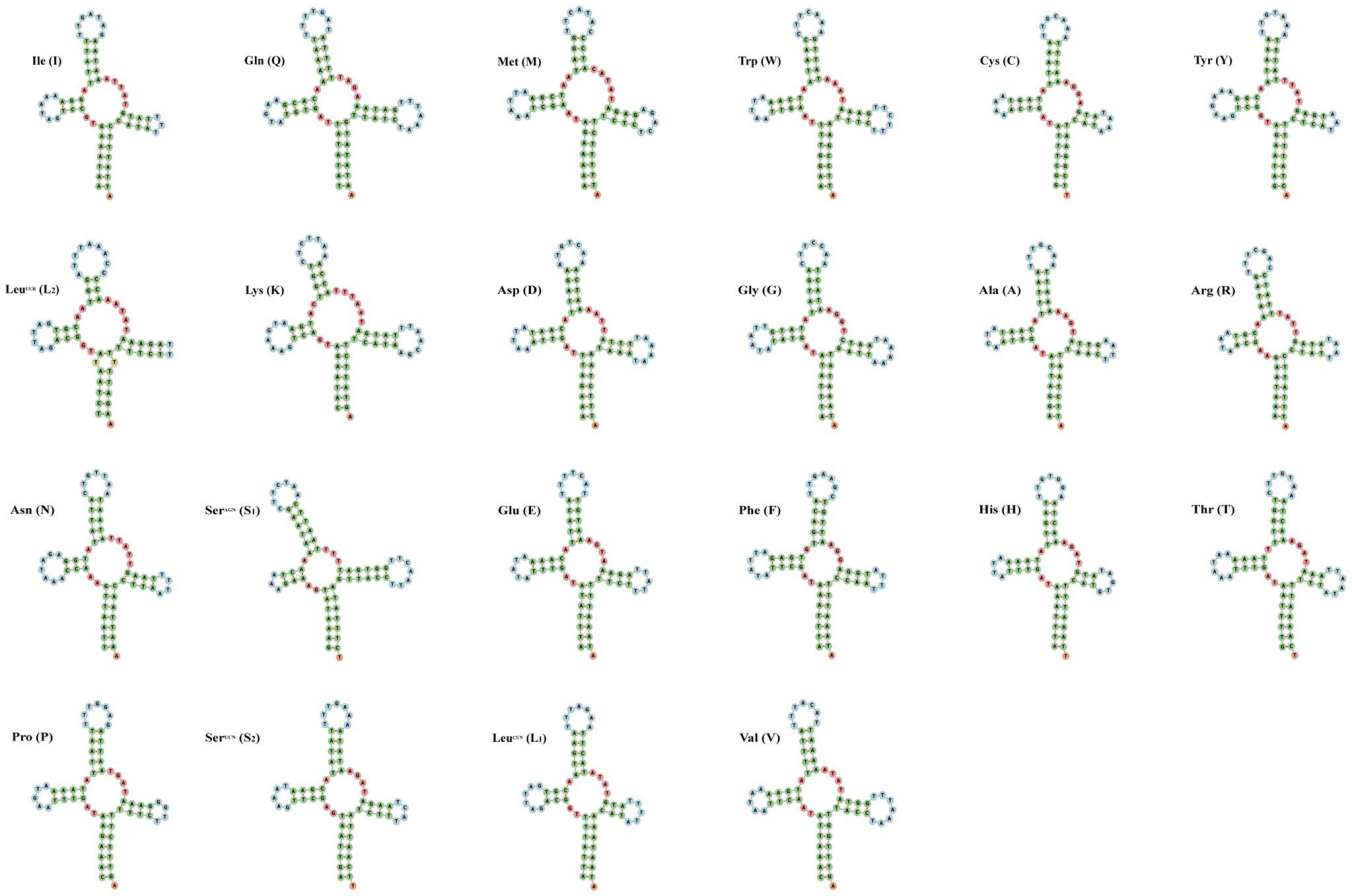
Putative secondary structure of the 22 mt tRNA of *Stenischia humilis*.

The small subunit of rRNA gene (rrnS) was situated next to trnV, and the large subunit of rRNA gene (rrnL) was located between trnL1 and trnV (Table 3). The length of rrnS and rrnL genes in *C. quadratus* were 783 and 1,250 bp, respectively, and the A + T contents of the rrnS and rrnL were 80.72 and 81.52%, respectively (Tables 3, 4). In the same way, the rrnS and rrnL genes of *S. humilis* were 785 and 1,266 bp, respectively, and the A + T contents of the rrnS and rrnL were 81.02 and 81.52% (Tables 3, 4). The length of 22 tRNA genes of *C. quadratus* ranged from 60 to 69 bp, and those of *S. humilis* ranged from 61 to 70 bp (Table 3). Most of the predicted secondary structures of 22 tRNA genes showed typical cloverleaf structure (Figures 3, 4).

### Phylogenetic analysis

**Figure 5:**
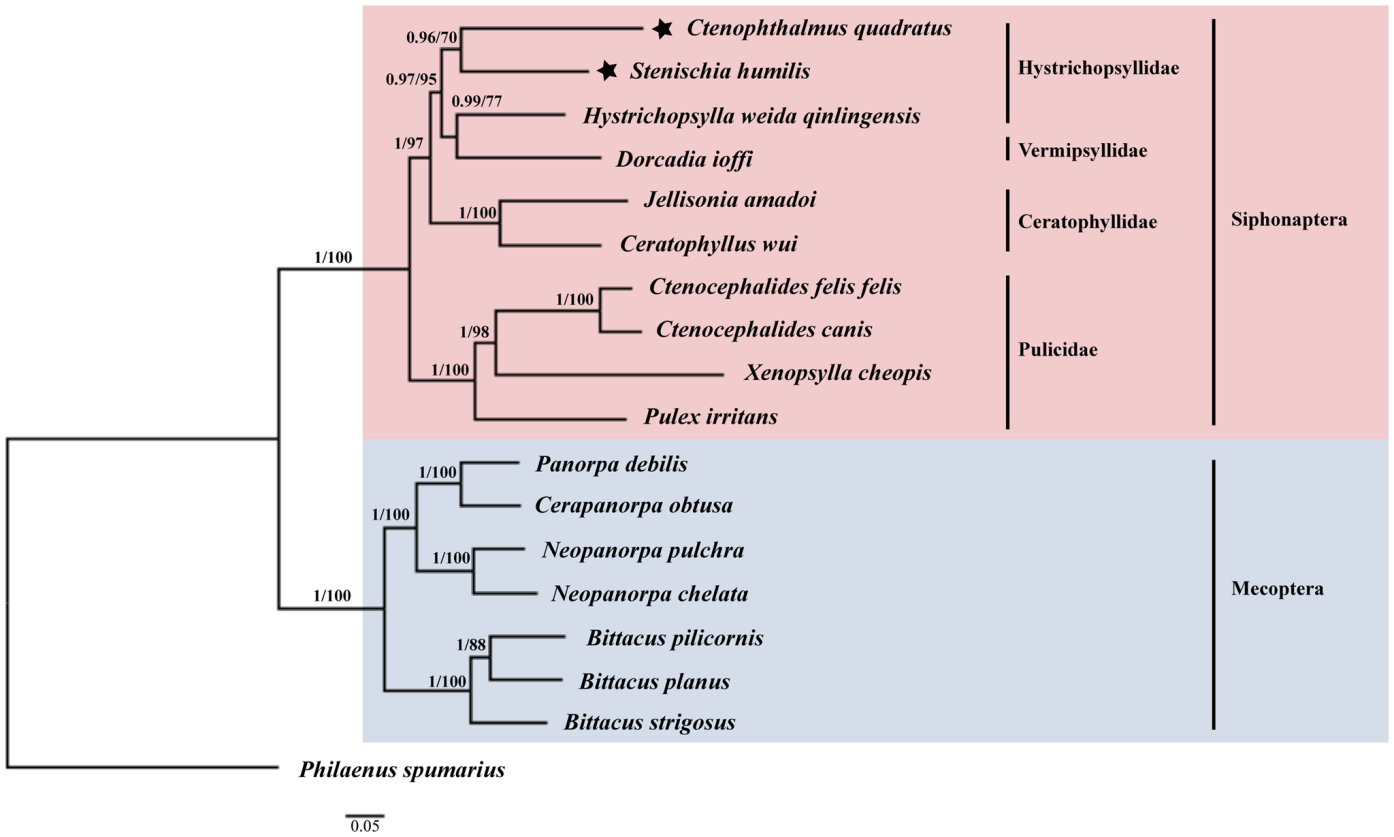
Phylogenetic relationships of 17 species of Siphonaptera and Mecoptera inferred from BI and ML analyzes of deduced nucleotide sequences of 13 PCGs. Bayesian posterior probability (Bpp) and Ultrafast bootstrap approximation (UFBoot) values were indicated at nodes, respectively. *Philaenus spumarius* (AY630340) was used as the outgroup.

Among the 17 species used for phylogenetic analysis by the BI and ML methods in this study, 10 species belonged to the Siphonaptera and seven species belonged to the Mecoptera (Figure 5). The monophyly of the order Siphonaptera and Mecoptera were strongly supported with the Bayesian posterior probability (Bpp) of 1 and the Ultrafast bootstrap approximation (UFBoot) of 100% in the BI and ML analyzes, respectively. Meanwhile, the family Hystrichopsyllidae may be paraphyletic. The close relationship between *C. quadratus* and *S. humilis* was strongly supported in BI analysis (Bpp = 0.96) and moderately supported in ML analysis (UFBoot = 70%). The close relationship between *Dorcadia ioffi* and *Hystrichopsylla weida qinlingensis* was strongly supported in BI analysis (Bpp = 0.99) and moderately supported in ML analysis (UFBoot = 77%). Nevertheless, *C. quadratus + S. humilis* was the sister group of *Dorcadia ioffi* + *Hystrichopsylla weida qinlingensis*, with strongly support in the BI and ML analyzes (Bpp = 0.97, UFBoot = 95%).

## Discussion

Fleas (Order Siphonaptera) are the most common blood-sucking ectoparasites and vector of many pathogens. People living in plague foci are more likely to be exposed to fleas carrying *Yersinia pestis* (32). The accurate identification and differentiation of flea species is significance for the control and diagnosis of flea-borne diseases (33). However, morphological identification of related and variant flea species are often challenging (11). Molecular data on fleas is still deficient. In the present study, the whole mt genomes of *Ctenophthalmus quadratus* and *Stenischia humilis* were analyzed for the first time to provide additional molecular data for phylogenetic studies of fleas.

In this study, the mt genomes of *C. quadratus* and *S. humilis* were consistent with the basic structural characteristics of other fleas, both containing 37 genes, two rRNA genes, 22 tRNA genes, 13 PCGs, and non-coding regions, and the gene arrangement sequences were consistent with that of other fleas (34). Negative AT-skew and GC-skew were found in their mt genomes, showing a bias toward T and C in nucleotide composition. Flea species are abundant, but few flea species have the complete mt genome in NCBI. So far, there are still less than 20 complete mt genomes in fleas, and there is no information on the *C. quadratus* and *S. humilis* mt genomes. Thus, the two mt genomes provided in this study will promote future studies on flea phylogeny and population evolution.

The monophyly of the Holometabola is well supported by morphological traits and molecular evidence (35, 36). Among them, the monophyly of Mecoptera remains extremely controversial. Phylogenetic analyzes using the 18S and 28S rRNA sequences suggest that the order Mecoptera may be paraphyletic, and that the order Siphonaptera (fleas) may be subordinate within Mecoptera (36–38). Similarly, the most recent study used the largest molecular dataset of over 1,400 protein-coding genes, as well as the smaller mitochondrial genome of 16 genes, indicate that Siphonaptera is nested within Mecoptera and suggest that Siphonaptera be treated as an infraorder of Mecoptera (39). Nevertheless, phylogenetic relationships inferred from 1,478 protein-coding genes strongly support that the Mecoptera and Siphonaptera are monophyletic (40). Phylogenetic analysis of 11 orders of holometabola using 13 PCGs in mitochondrial genomes supported the monophyly of Siphonaptera and paraphyly of Mecoptera. The results show that the Siphonaptera is an independent order and as a sister group of the family Boreidae, rather than subordinate to Mecoptera (34). The above results show that the phylogenetic position of fleas in holometabolan insects is still unclear and highly controversial.

This study is the first in the world to analyze the mt genomes of *Ctenophthalmus quadratus* and *Stenischia humilis*, and to analyze their phylogenetic positions in fleas using 13 PCGs. The results support that the order Siphonaptera is monophyletic. And the family Hystrichopsyllidae is paraphyletic. The seven species of Mecoptera analyzed in this study cluster together to form one clade, and the 10 species of Siphonaptera cluster together to form another clade, and strongly support a sister relationship between the orders Siphonaptera and Mecoptera, consistent with other research findings (34). Up to now, there is no information on the mt genomes of the genera of Ctenophthalmus and Stenischia species. The present study is the first to analyze flea species from the two genera. Due to the lack of mt genome data for all lineages of fleas, which is not fully representative of the overall phylogenetic relationships of fleas. Hence, further acquisition of mt genomes from more flea species is needed to further evaluate the evolutionary relationship between the orders Siphonaptera and Mecoptera, and their phylogenetic position in holometabolous insects with more comprehensive molecular data.

## Conclusion

The present study is the first to obtained the complete mitochondrial genomes of *Ctenophthalmus quadratus* and *Stenischia humilis*. Phylogenetic analysis of eight other fleas and seven species of Mecoptera demonstrated that the *C. quadratus* and *S. humilis* are distinct species in the same family, and provided a sister relationship between the Siphonaptera and Mecoptera, supporting the monophyly of fleas. These mt genomes provide a hint for the phylogenetic position of *C. quadratus* and *S. humilis* in fleas, and provide novel genetic information for the phylogeny and evolution of fleas.

## Data availability statement

The datasets presented in this study can be found in online repositories. The names of the repository/repositories and accession number(s) can be found at: https://www.ncbi.nlm.nih.gov/genbank/, OQ023577; https://www.ncbi.nlm.nih.gov/genbank/, OQ366410.

## Ethics statement

The animal study was approved by the Administration Committee of Experimental Animals, Dali University. The study was conducted in accordance with the local legislation and institutional requirements.

## Author contributions

XY and BC designed the study. D-dJ and X-yL are in charge of data gathering. Y-fL and BC designed the primers and conducted the experiment. BC and XW collated and analyzed the data. G-pY and Q-fZ contribute to the results discussion. BC wrote the manuscript. Y-fL, X-yL, D-dJ, XW, Q-fZ, G-pY, and XY writing--review and editing. All authors read and provided critical viewpoint and determine the final version.

## Funding

This study was funded by the following organizations and initiatives: Yunnan Natural Science Foundation (2017FD139), and Scientifc Research Fund of Yunnan Education Department (2022 J0687).

## Conflict of interest

The authors declare that the research was conducted in the absence of any commercial or financial relationships that could be construed as a potential conflict of interest.

## Publisher’s note

All claims expressed in this article are solely those of the authors and do not necessarily represent those of their affiliated organizations, or those of the publisher, the editors and the reviewers. Any product that may be evaluated in this article, or claim that may be made by its manufacturer, is not guaranteed or endorsed by the publisher.
